# Monthly Summary

**Published:** 1878-09

**Authors:** 


### MONTHLY SUMMARY.

---

*The Impurities and Tests of Chloroform.*—A. H. Mason says: The impurities or foreign ingredients which have been detected hitherto are alcohol in excess, aldehyde, ether, hydrochloric acid, hypochlorous acid, and empyreumatic or chlorinated oils. The presence of the two former is easily detected by physical and chemical means. The acids would be present owing to decomposition, or with the chlorinated oils, owing to imperfect purification.

To detect the presence of alcohol, if present, the addition of a little chromic acid will cause the chloroform to turn green. Dr. Davy states that if a little of the suspected substance is heated with a solution of molybdic acid in sulphuric acid, it turns blue, if alcohol be present, and as he found all samples of English manufacture were affected by that test, he assumed that all English chloroform is adulterated. According to Roussin, pure chloroform, shaken up with dinitrosulphide of iron, remains colorless, but if it contains alcohol, ether, or wood spirit, it acquires a dark color. A few years ago I applied this test, but without success, although contaminated specimens were experimented upon, and to ensure the purity of the salt I got some manufactured by an eminent firm of manufacturing chemists. The presence of acids or of chlorinated oils will be evident from the disagreeable odor. In the administration of chloroform the presence of these chlorinated compounds to any appreciable extent produces a marked effect upon the system. They occasion a peculiar throbbing headache, and a rapid prostration of the vital powers. These symptoms may be observed when the chloroform is inhaled only for a short time; and there can be no doubt that they are very often the causes of the discomfort so often resulting from the free use of impure samples of this anæsthetic.—*Chemist and Druggist.*

*Treatment of Furuncles by Arnica.*—Dr. Planat, of Nice, claims that arnica has the power of aborting an eruption of boils, except when due to diabetes, with extraordinary rapidity. His method of employing it is very simple. In order to render its action on the small vessels more energetic, he applies it directly to the inflamed spot in the form of an ointment, of which the formula is as follows: Extract of fresh arnica flowers, ʒ i jss., honey, ʒ v. If the mixture be too fluid, he adds powdered lycopodium or althææ, or some other inert powder, until it acquires the proper consistency. It is then spread pretty thickly on a bit of oiled silk or diachylon plaster, and applied directly to the boil. It is rarely necessary to renew the dressing more than once in twenty-four hours. As a rule, two or three dressings are enough to make the furuncle abort, no matter what be the period of its evolution.

A curative action is also obtained by the internal administration of the drug. Dr. Planat gives from three to four drops of the tincture, largely diluted, every two hours, and he has seen the furuncular eruption disappear very rapidly under the treatment.—*Jour. de Med., etc., de Bruxelles.*

---

*Theropeutic Value of Nitrite of Lead.*—The late Dr. Madison Marsh, of La., a few years ago urged upon the profession, through the columns of this journal, the very great value of nitrite of lead in many skin affections and superficial erosions. Some recent observations of an Italian physician, Dr. Galletti, add further evidence on this point. This writer states that he has recently effected a cure in three cases of epithelioma, in one of which the part affected was the nose, in a second the cheek, and in the third the sternum. The mode in which he applied the remedy was by dusting the powder over the affected part, and recovery took place when this had been done about four times. Two obstinate ulcers of the foot, which had proved rebellious to other methods, quickly recovered under the same treatment. Dr. Vanzetti has recently recommended the use of the nitrite of lead in onychia maligna.—*Med. and Surg. Reporter.*

---

*Stilbeine, a Preparation for Cleansing Instruments*—This handy preparation is simply a combination of rubber and impalpable emery powder. It comes in tablets of the shape and size of the ordinary rubber eraser. It removes rust and blood-stains, etc., immediately, and without scratching or soiling the instrument. All that is necessary is to rub the instrument with the composition, when it rapidly acquires a brilliant polish.—*Annee Med.*

*Skin Grafting in the Colored Races.*—A French naval Surgeon, Dr. Maurel, stated at a scientific meeting in Paris, that during a two years' residence at Guiana, he had made numerous experiments on epidermic transplantation, placing the graft on persons of different race and color. He found that not only did the graft take well, whatever description of transplantation was made—whether transported from the skin of a black to that of a white, or the reverse—but that there always remained a whitish line at the point of junction, wherein pigmentation was not produced. The pigment disappeared when a graft was transplanted from a black to a white person ; but when the two individuals were highly colored, the graft remained black, except at the point of cicatrization.—*Med. and Surg. Reporter.*

---

*Death under Ether.*—A coal-porter, over fifty years of age, was admitted into the London Hospital for strangulated hernia last week. He was a well-developed man, and had been the subject of inguinal hernia more than three years, but till the last week had always been able to restrain it, and had never worn a truss. He had never experienced any special inconvenience from the rupture till four days before his admission to the hospital, when he was troubled with abdominal pain, complete obstruction of the bowels, and frequent vomiting, latterly becoming fæcal. His general health had been failing in an increasing degree during the last few years, so that he had sought medical advice. When admitted to hospital, he complained much of abdominal pain, and was unable to reduce his rupture. When examined, he presented a large scrotal hernia, somewhat tense. During the examination, he began vomiting fecal matter. Taxis having been used, but the symptoms remaining unrelieved, it was considered advisable to place the man under an anæsthetic. The house-surgeon accordingly administered ether, using not more than an ounce and a half in all. The patient came under the influence of the ether rapidly, and without difficulty or adverse symptoms. The local examination was then proceeeded with. The patient's repirations were perfectly regular, and his pulse good, when, about six minutes after inhalation began, a sudden spasmodic inspiratory sound was heard, as if he were choking. His tongue was immediately drawn forward with the forceps ; but respiration had ceased, although his pulse continued to beat for another half minute. Silvester's artificial respiration was employed, but no spontaneous inspiratory effort followed. During the artificial respiration, some fecal matter came up into the mouth. These efforts continued a quarter of an hour, but proved useless. At the *post-mortem* examination, the left ventricle was found contracted ; but the heart appeared healthy. The lungs were extremely congested. There was fecal staining of the œsophagus and larynx, but no such matter had been drawn into the lungs. The liver was healthy. The kidneys were slightly granular, but not congested. The portion of small intestine which had been strangulated was about twenty inches in length, extremely congested, with commencing peritonitis upon its surface. It must be remembered that in such a case as this, where strangulation had existed four days and was attended with constant vomiting and a feeble circulation, there were many circumstances predisposing to death besides the administration of ether. —*Med. News.*

---

*The Antiseptic and Therapeutic Properties of Boracic Acid.*— The *Lancet* states that G. Polli has reported, at a recent meeting of the Academy of Sciences, of Lombardy, the results of numerous researches in which beer, meat, eggs, blood, and urine were treated with boracic acid and borax for thirty days, during the summer time, and were found still to retain their freshness, and to present no traces of fermentation having taking place in them. In control experiments, on the other hand, without the addition of the salt, but in some instances, with the additton of sulphate of soda, the fluids passed into a state of complete decomposition in the course of fifteen days. The energetic disinfecting power possessed by boracic acid and borax, and the facility with which these substances can be absorbed into the economy, led Polli to recommend their employment in diseases in regard to the infectious nature of which no doubt exists, or in which septic conditions readily arise. He adduces several examples in which the fubrile conditions of tuberculosis underwent diminution. No benefit was obtained by Professor Visconti from experiments made with these remedies in malaria, though other observers have arrived at a different conclusion. In chronic cystitis the muco-purulent discharge quickly diminished, and even altogether disappeared in the course of a few days, and rapid improvement occurred in cases of bad suppurating wounds when they were applied externally. The dose recommended by Polli, is 75 grains of boracic acid and 150 grains of borax per diem.— *Med. and Surg. Reporter.*

---

*Vermilion.*—Vermilion is a mixture of sulphur and mercury, and is frequently found to turn to a dark brown color if exposed to the atmosphere. A remedy for this is said to be to add one-eighth part flour of sulphur to the paint when mixing. To detect adulteration in vermilion, place a little on a red hot iron; if pure, it will evaporate entirely; if not, there will be an earthy residue.—*Druggists Circular.*

